# Transcriptional Profiling of Newly Generated Dentate Granule Cells Using TU Tagging Reveals Pattern Shifts in Gene Expression during Circuit Integration^1^,^2^

**DOI:** 10.1523/ENEURO.0024-16.2016

**Published:** 2016-03-07

**Authors:** Christina Chatzi, Yingyu Zhang, Rongkun Shen, Gary L. Westbrook, Richard H. Goodman

**Affiliations:** Vollum Institute, Portland, Oregon 97239

**Keywords:** hippocampus, nascent RNAs, neural development, neurogenesis, transcriptome

## Abstract

Despite representing only a small fraction of hippocampal granule cells, adult-generated newborn granule cells have been implicated in learning and memory (Aimone et al., 2011). Newborn granule cells undergo functional maturation and circuit integration over a period of weeks. However, it is difficult to assess the accompanying gene expression profiles *in vivo* with high spatial and temporal resolution using traditional methods. Here we used a novel method [“thiouracil (TU) tagging”] to map the profiles of nascent mRNAs in mouse immature newborn granule cells compared with mature granule cells. We targeted a nonmammalian uracil salvage enzyme, uracil phosphoribosyltransferase, to newborn neurons and mature granule cells using retroviral and lentiviral constructs, respectively. Subsequent injection of 4-TU tagged nascent RNAs for analysis by RNA sequencing. Several hundred genes were significantly enhanced in the retroviral dataset compared with the lentiviral dataset. We compared a selection of the enriched genes with steady-state levels of mRNAs using quantitative PCR. Ontology analysis revealed distinct patterns of nascent mRNA expression, with newly generated immature neurons showing enhanced expression for genes involved in synaptic function, and neural differentiation and development, as well as genes not previously associated with granule cell maturation. Surprisingly, the nascent mRNAs enriched in mature cells were related to energy homeostasis and metabolism, presumably indicative of the increased energy demands of synaptic transmission and their complex dendritic architecture. The high spatial and temporal resolution of our modified TU-tagging method provides a foundation for comparison with steady-state RNA analyses by traditional transcriptomic approaches in defining the functional roles of newborn neurons.

## Significance Statement

Unlike most regions of the brain, the hippocampus produces new neurons well into adulthood. These newborn neurons contribute to learning and memory, presumably because they functionally integrate into pre-existing circuits, whereas their decrease during aging may be associated with cognitive decline. This process occurs by dynamic regulation of many genes as these cells interact with their environment. We adapted an *in vivo* approach, providing both temporal and spatial control of gene expression that captures the transcriptional profiles of discrete populations of newborn neurons at distinct stages of their maturation. Our study provides insights into the underlying pathways controlling hippocampal newborn neuron differentiation and functional integration, which could lead to new approaches for enhancing their production, integration, and survival.

## Introduction

The subgranular zone (SGZ) of the adult hippocampal dentate gyrus (DG) is one of two neurogenic niches in the mammalian brain where new neurons are continuously generated throughout adulthood (Taupin and Gage, 2002; van Praag et al., 2002; Toni et al., 2008; Zhao et al., 2008). Adult-generated newborn hippocampal neurons ultimately integrate into pre-existing functional circuits, where, in mice as well as in humans, they may have specific roles in learning and memory (Gould et al., 1999; Clelland et al., 2009; Deng et al., 2010; Sahay et al., 2011). How newborn neurons are incorporated into an existing circuit is poorly understood and perhaps even more remarkable than their generation in the first place. Although the differentiation of newborn granule cell has been extensively analyzed (Overstreet-Wadiche and Westbrook, 2006), the patterns of gene expression underlying distinct stages of adult-generated newborn granule cell growth and maturation remain largely unknown. Studies of steady-state levels of mRNA in newborn neurons *in vivo* have been limited by the vast excess of other cell types and the dynamic changes in the phenotype of new neurons (Gould et al., 1999; Jagasia et al., 2009; Kuwabara et al., 2009; Mu et al., 2010; Fuentealba et al., 2012; Bracko et al., 2012). Thus, we lack an understanding of the dynamics of gene networks during adult newborn hippocampal neuron development, which requires *in vivo* approaches of mRNA expression that provide high temporal and spatial resolution.

The integration of newborn granule cells can be divided into a stage of immature neurons (∼2 weeks postmitosis) and integrated mature neurons (≥4 weeks postmitosis; van Praag et al., 2002; Overstreet-Wadiche and Westbrook, 2006). By 2 weeks postmitosis, adult-generated hippocampal granule cells have extended primary dendrites into the inner molecular layer and nascent axons to stratum lucidum in CA3. These cells have the properties of immature neurons with broad action potentials mediated by Na^+^ channels and receive spontaneous depolarizing GABAergic activity, but almost no excitatory glutamatergic synapses (Ge et al., 2006; Overstreet-Wadiche and Westbrook, 2006). Within the next week, they elaborate spiny dendrites that reach the middle and outer molecular layer, and begin to receive functional glutamatergic inputs from the entorhinal cortex such that surviving newborn neurons have nearly mature phenotypes by 4 weeks postmitosis (van Praag et al., 2002; Overstreet-Wadiche and Westbrook, 2006).

We hypothesized that the distinct stages of newborn neuron maturation are associated with characteristic patterns of gene expression. The convergence of stimuli that shape the integration of newborn neurons into functional networks implies the participation of many genes. Because many of these genes may turn over rapidly, this places a premium on understanding the cellular specificity, as well as the timing and sequence of gene expression. Measurements of steady-state mRNAs cannot easily resolve these issues. To address these issues, we used a novel viral-mediated thiouracil (TU)-tagging method (Miller et al., 2009), combined with RNA sequencing (RNAseq) to compare gene expression patterns of the small subset of adult-generated immature granule cells with mature granule cells in the adult dentate gyrus *in vivo*.

## Materials and Methods

### Animal protocols

All procedures were performed according to the National Institutes of Health *Guidelines for the Care and Use of Laboratory Animals*, and were in compliance with approved institutional animal care and use committee (IACUC) protocols at Oregon Health & Science University. Subjects were young adult, female, 6-week-old C57BL/6J mice (The Jackson Laboratory).

### Virus production and stereotaxic injections

Moloney Murine Leukemia Virus-based retroviral vectors require cell mitosis for transduction, and were used to identify and manipulate adult-born granule cells. Uracil phosphoribosyltransferase (UPRT)-expressing retroviruses were created using a pSie-based viral backbone, and expression was driven by a ubiquitin promoter and followed by a woodchuck post-transcriptional regulatory element (van Praag et al., 2002; Tashiro et al., 2006). UPRT-expressing FUGW lentiviral vector was created as described (Lois et al., 2002; Luikart et al., 2011a). Mice were anesthetized using an isoflurane gas system (Veterinary Anesthesia Systems), placed in a Kopf stereotaxic frame fitted with a gas nose cone, and a skin incision was made and holes were drilled at *x* (±1.1 mm from bregma) and *y* (−1.9 mm from bregma). Using a 10 µl Hamilton syringe with a 30 gauge needle and the Quintessential Stereotaxic Injector (Stoelting), 2 μl of mixed viral stock (1 μl of each virus) was delivered at 0.25 µl/min at *z*-depths of 2.5 and 2.3 mm. The syringe was left in place for 1 min after each injection before being withdrawn slowly. The skin above the injection site was closed using veterinary glue. Animals received postoperative lidocaine and drinking water containing children's Tylenol.

### Immunohistochemistry and quantitation

Mice were terminally anesthetized according to IACUC-approved protocols, transcardially perfused with saline and 20 ml of 4% paraformaldehyde, and brains were postfixed overnight. Coronal sections (100 μm thick) of the hippocampus were collected from each mouse and permeabilized in 0.4% Triton in PBS (PBST) for 30 min. Sections were then blocked for 30 min with 5% horse serum in PBST and incubated overnight (4°C) with primary antibody in 5% horse serum/PBST. The primary antibodies used were as follows: anti-doublecortin (DCX; 1:600; Millipore); anti-glial fibrillary acidic protein (GFAP; 1:1000; Dako); anti-Calbindin D-28K (1:500; Chemicon); anti-NeuN (1:500; Sigma-Aldrich); anti-nestin (1:100; Chemicon), anti-CD68 (1:500; Abcam); and anti-Olig2 (1:300; Abcam). After extensive washing, sections were incubated with the appropriate secondary antibody conjugated with Alexa Fluor 488, 568, or 647 (Molecular Probes), for 2 h at room temperature. They were then washed in PBST (2× 10 min) and mounted with DAPI Fluoromount-G (SouthernBiotech).

For quantification of immunopositive cells, six sections offering dorsal-to-ventral coverage of the dentate gyrus were stained from each animal. Three to five animals were analyzed for each time point and condition. Slides were coded and imaged by an investigator who was blinded to experimental condition with a Zeiss LSM780 confocal microscope under a 10× 0.45 numerical aperture (NA), 20× or 40× 0.8 NA lens. A 49 μm z-stack (consisting of seven optical sections of 7 μm thickness) was obtained from every slice. Positive cells were counted per field from every *z*-stack using ImageJ software, averaged per mouse, and the results were pooled to generate mean values.

### Nascent mRNA labeling and sequencing of dentate gyrus granular neurons

Using a stereotaxic apparatus, 1.25 µl of 1 mm 4-TU (Sigma-Aldrich) was delivered intracerebroventricularly into the dorsal third ventricle (−1.9 mm posterior to bregma, ±0.0 mm lateral to midline, −2.25 ventral to the skull surface) at a rate of 0.1 µl/min. Because *in vitro* preliminary data indicated that maximum TU incorporation peaked at 16-18 h (data not shown), dentate gyri from individual mice were harvested after overnight TU exposure and stored in 1 ml of TRIzol. RNA was extracted using the RNeasy kit (Qiagen) with a modified protocol. Briefly, after tissue homogenization with TissueRuptor (Qiagen) in TRIzol, 200 µl of chloroform was added to each sample and mixed thoroughly. Samples were spun down, and supernatants were transferred to new collection tubes. Five hundred microliters of 100% ethanol was then added to each sample, and tubes were inverted several times to mix samples well. The mixture was passed through an RNeasy column, and columns were washed two times with RPE buffer. Columns were dried by high-speed spin, and RNA was eluted with 80 µl of water. Samples were then treated with DNAse I for 15 min, processed using an RNeasy MinElute kit, and eluted in 13 µl of RNase-free H_2_O. Samples were then heated to 65° for 5 min and centrifuged at 12,000 rpm for 1 min and transferred to chilled collection tubes containing 12.5 µl of ice-cold 2× binding buffer (EDTA Tris-HCL, pH 7.4, NaCl 2 m, Tween 0.1%). Four microliters of Myone C1 streptavidin magnetic beads (LifeScience Technology) was blocked with yeast tRNA and suspended in 25 µl 1× binding buffer (EDTA Tris-HCl, pH 7.4, NaCl 1 m, Tween 0.05%). Twenty-five microliters of chilled suspended beads were added into each sample and mixed well. Samples were incubated on ice for 10 min and then incubated at room temperature for up to 1 h with constant shaking. Beads were collected using a magnetic stand and washed with 1× binding buffer at room temperature twice for 2 min with constant shaking. Then samples were washed at 55° with 1× binding buffer twice for 30 s each time, and with 1× TE buffer twice briefly. After washes, RNA was released from beads by adding 10 µl of 100 mm DTT and incubation for 10 min. Five microliters of RNA was used for cDNA amplification using a SMART-Seq Ultra Low Input RNA Kit for Sequencing (Clontech). Amplified cDNA was fragmented with a Covaris ultrasonicator and RNAseq libraries were prepared using a TruSeq RNA Library Prep Kit (Illumina). High-throughput sequencing was performed on a Hiseq2000 platform, and six to eight samples were mixed in a single lane for each run. The sequencing quality of all the samples was checked by FastQC (http://www.bioinformatics.babraham.ac.uk/projects/fastqc/). Tophat (Trapnell et al., 2010) was used to map the sequencing reads against mouse mm10 genome assembly and annotation. The uniquely mapped reads were assigned to and counted in each individual gene by HTSeq-count (Anders et al., 2015) according to their coordinates. The data quality of the count data was examined by principal component analysis (PCA) and density distribution plotting. The genes of zero or very low expression level across all the samples were filtered out to minimize the interference of those genes in the following analysis and to increase the statistical detection power (Cambronne et al., 2012). The remaining genes were normalized by a geometry median approach, which was implemented in DESeq. We used ANOVA to determine which mRNAs were significantly enriched in a pairwise manner between lentiviral and retroviral samples (Cambronne et al, 2012). We also used DAVID (Database for Annotation, Visualization and integrated Discovery) in combination with Gene Ontology (GO) or Kyoto Encyclopedia of Genes and Genomes (KEGG) databases for functional analysis of significant enriched mRNAs. Violin and density plots were generated by ggplot2, an R package. The UCSC Genome Browser was used for sequencing data visualization. Other tools and scripts used for data parsing, analysis, and visualization were written in R, Linux shell, or Perl (available upon request). All of the raw data will be uploaded into the National Center for Biotechnology Information GEO (Gene Expression Omnibus) database.

### Laser capture microdissection

Fresh-frozen coronal brain sections (12 µm) were collected by cryostat sectioning on polyethylene napthalate membrane slides, and were fixed for 1 min in 75% ethanol, followed by staining for 1 min with Cresyl Violet acetate (1 mg/ml; Sigma-Aldrich) and a final wash in 100% ethanol for 1 min. Cell populations from either the subgranular zone or the granular cell layer (GCL) of the dentate gyrus were laser captured using a Leica Microsystems laser microdissection system under an HC PL FL L 40×/0.60 XT objective (Leica Microsystems) and collected in lysis buffer (50 µl; RLT buffer, Qiagen). Total RNA from laser capture microdissection (LCM) samples of the SGZ and GCL was isolated on an individual animal basis (*n* = 5) and extracted using an RNeasy Micro Kit (Qiagen). RNA was amplified linearly using SMART-Seq Ultra Low Input RNA Kit for Sequencing library prep kit (Clontech).

### Quantitative PCR of laser capture tissue samples

RNA from laser-captured samples was extracted using the RNeasy Micro Kit. Genomic DNA was removed by on-column DNase I digestion. RNA amount and quality was monitored by an Agilent bioanalyzer. Five hundred picograms of RNA was used for cDNA amplification using the SMART-Seq Ultra Low Input RNA Kit (Clontech). Quantitative PCR was performed using amplified cDNA on an Opticon Real-Time PCR machine. iQ qPCR (Bio-Rad) mastermix was used for all of the reactions. RNA expression levels were normalized to β_2_-microglobulin mRNA levels. Primer specificity and genomic RNA contamination was monitored using the melting curve of the PCR products. Primer sequences were obtained from the Origene validated quantitative PCR (qPCR) primer collection (Origene Technologies; Table 1). Statistical analysis was performed with Prism software (GraphPad Software). The two groups were compared using an unpaired nonparametric Mann–Whitney test, and significance was defined as *p* < 0.05 (*).

**Table 1:** Primer sequences

Lrrc8b-F	CCA TCT GAC CTT CAT TCC CGA G	Lrrc8b-R	TCC CAG GAG TAG ACA CTG AAG C
nova2-F	AAG CCT GAG GTG GTC AAC ATC C	nova2-R	GAC TGT TCC ATC ACC GCC TTC A
onecut2-F	TTC CAG CGC ATG TCT GCC TTA C	onecut2-R	GAA GAT GGC GAA GAG TGT TCG G
dusp8-F	CTT ATC CAG CCT GCT ACA CGG A	dusp8-R	AGC TTG CTG AGC AGG ATG GAC A
Prox1-F	CTG AAG ACC TAC TTC TCG GAC G	Prox1-R	GAT GGC TTG ACG CGC ATA CTT C
Kcnq3-F	AAG CCT ACG CTT TCT GGC AGA G	Kcnq3-R	ACA GCT CGG ATG GCA GCC TTT A
Dpysl2-F	GAC CAT CTC TGC CAA GAC ACA C	Dpysl2-R	GGA ATG TAG CGT CCT GAG CCT T
Mycbp2-F	CCT ACT GTG CAA ACT GGA CTC C	Mycbp2-R	CTT CGG CTT GAC TAG CTG AGT C
Pex5l-F	ATG AGC AGG CAG CTA TTG TCG C	Pex5l-R	CTT CAG TGC CTC ACA AGC ATC C
Ppp1r9a-F	TGC CCA GTA TGA TGC TGA CGA C	Ppp1r9a-R	ATG TCC TCG TTC TCA GGC AGC T
Robo2-F	CCA CCA TCC AAA CCT CAG GTC A	Robo2-R	TCT GCC AGC TAT TGC TCA CCG A
Slc27a4-F	GAC TTC TCC AGC CGT TTC CAC A	Slc27a4-R	CAA AGG ACA GGA TGC GGC TAT TG
Ankhd1-F	CTG TTT CCA GGG TCG AGC AGA A	Ankhd1-R	CTT CCA ACC TCT GCA TAT CCT CC
Ankrd17-F	GCA GCA AAT GGT GGA CAC CTA G	Ankrd17-R	CTA AGT AGC GCA CCA CCT TCA C
Eif2c1-F	CTG CCT TCT ACA AAG CAC AGC C	Eif2c1-R	TCT GTC CAC AGT GGG TCA CTT C
Eif2c3-F	CTT CTG TGT TCC AGC AAC CAG TG	Eif2c3-R	GGC ACA GTA TCT GCT TGG ATG G
Eif2c4-F	CAC ACG CAT CAT CTA CTA CCG C	Eif2c4-R	GCC GAT AGT CTT CCT CCA AGC T
Hnrnpa2b1-F	CGG TGG CAA TTT TGG ACC AGG A	Hnrnpa2b1-R	CCA TAA CCA GGG CTA CCT CCA A
Tjp1-F	GTTGGTACGGTGCCCTGAAAGA	Tjp1-R	GCTGACAGGTAGGACAGACGAT
Dscam-F	CATCCGCATGTACGCCAAGAAC	Dscam-R	GAGATGAGGTGGGTTCCAAGTG
Cadm1-F	ACTTCTGCCAGCTCTACACGGA	Cadm1-R	CCCTTCAACTGCCGTGTCTTTC
Chl1-F	GGAAAAGCCGTCATCACAGCGA	Chl1-R	GTGAGTCACACTGGCTTTCGCA
Sorbs1-F	TATCAGCCTGGCAAGTCTTCCG	Sorbs1-R	CCCGTCTGATTCCCTCTTCACT
Ncam1-F	GGTTCCGAGATGGTCAGTTGCT	Ncam1-R	CAAGGACTCCTGTCCAATACGG
Elavl3-F	TGC AGA CAA AGC CAT CAA CAC CC	Elavl3-R	CCA CTG ACA TAC AGG TTG GCA TC
Prox1-F	CTG AAG ACC TAC TTC TCG GAC G	Prox1-R	GAT GGC TTG ACG CGC ATA CTT C
Elavl3-F	TGC AGA CAA AGC CAT CAA CAC CC	Elavl3-R	CCA CTG ACA TAC AGG TTG GCA TC
B2m-F	ACAGTTCCACCCGCCTCACATT	B2m-R	TAGAAAGACCAGTCCTTGCTGAAG

## Results

### *In vivo* pulse-chase detection of nascent mRNAs by TU tagging

**Figure 1.**
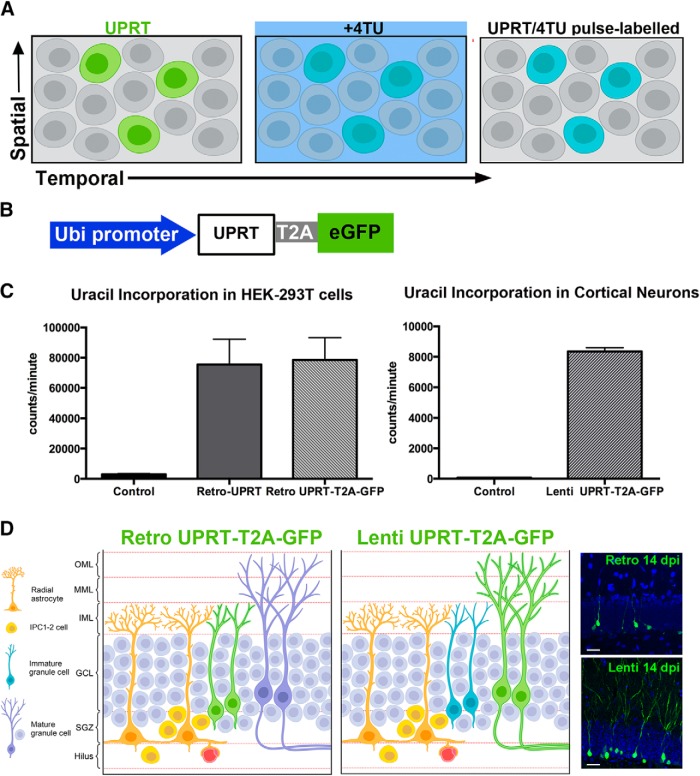
TU tagging in the adult DG. ***A***, Overview of TU-tagging method: UPRT cell type specificity confers spatial control (green), and pulse chase of 4-TU provides temporal control (blue). ***B***, Construct for virus-mediated UPRT expression. ***C***, Uracil was incorporated in HEK-293 T cells transduced with UPRT-expressing retroviruses (left) and in neonatal hippocampal neurons transduced UPRT-expressing lentivirus (right). Data are plotted as the mean ± SD. ***D***, Schematic of strategy for TU tagging of immature newborn and mature neurons with UPRT-expressing retroviruses and lentiviruses, respectively, in the adult DG (left and middle). Representative images of newborn and mature neurons that robustly express UPRT-T2A-GFP retrovirus (right top) and lentivirus (right bottom) at 14 DPI. Scale bar, 20 μm.

To investigate the differences in nascent mRNAs in adult-generated immature neurons 2 weeks postmitosis compared with mature granule cells, we refined the “TU-tagging” technique for use in the CNS *in vivo* (Cleary et al., 2005; Zeiner et al., 2008; Miller et al., 2009). This approach provides high spatial and temporal resolution of changes in gene expression. TU tagging allows cell type-specific biosynthetic labeling of actively transcribed RNA within intact tissues, which is not possible with traditional transcriptome analysis that measures steady-state RNA levels (Miller et al., 2009; Gay et al., 2013). TU tagging exploits the activity of a *Toxoplasma gondii* uracil salvage enzyme, UPRT, which is not present in mammalian cells, but exogenous expression can be targeted to specific cell types within intact tissue (Fig. 1*A*, left). Under normal conditions, UPRT converts uracil to uridine monophosphate. However, following pulse labeling with the modified analog 4-TU, thiol groups are incorporated into nascent RNA in UPRT-expressing cells within a narrow time window (Fig. 1*A*, middle and right; Cleary et al., 2005; Zeiner et al., 2008). Thiol labeling of nucleotides by UPRT has little or no effect on cellular physiology (Cleary et al., 2005), and TU-tagged RNAs then can be biotinylated, purified, and further processed for RNAseq.

To achieve cell type specificity of labeling in the adult dentate gyrus *in vivo*, we used viral constructs to target *UPRT* gene expression to granule cells. Virus-mediated cell manipulations are efficient tools for investigating molecular mechanisms involved in adult DG neurogenesis in an intact brain environment. For example, stereotaxic injections into the dentate gyrus of retroviral vectors, which are selective for dividing cells, have been used for birth dating and labeling adult newborn neurons (van Praag et al., 2002), whereas VSV-g pseudotyped lentiviral vectors show a tropism toward mature granule cells (Lois et al., 2002; Luikart et al., 2011a, 2011b). Thus, we designed matching retroviral and lentiviral vectors expressing a bicistronic reporter construct containing the coding sequence for *T. gondii* UPRT and green fluorescent protein (GFP) separated by a T2A peptide linker sequence under the control of the ubiquitin promoter (Fig. 1*B*), to allow high-level expression *in vivo*.

To determine the efficiency of the UPRT viral vectors, we tested ^3^H-uracil incorporation into RNA of HEK-293 T cells. Control cells lacking the UPRT transgene showed minimal ^3^H-uracil incorporation, whereas cells transduced with a retrovirus expressing UPRT or UPRT-T2A-GFP showed robust incorporation (Fig. 1*C*, left; control, 3,031 ± 435; retro-UPRT, 75,527 ± 16,645; retro-UPRT-T2A-GFP, 78,518 ± 14,473; *n* = 3). Additionally, primary cultures of neonatal hippocampal neurons infected with a lentivirus expressing UPRT-T2A-GFP demonstrated both UPRT activity (Fig. 1*C*, right; control, 72 ± 1; lenti-UPRT-T2A-GFP, 8,352 ± 245; *n* = 3), as assayed by ^3^H-uracil incorporation, and GFP expression as assayed by immunofluorescence (data not shown), indicating that viral expression of UPRT led to cell-specific uracil incorporation.

### Retroviral and lentiviral expression of UPRT-T2A-GFP

**Figure 2.**
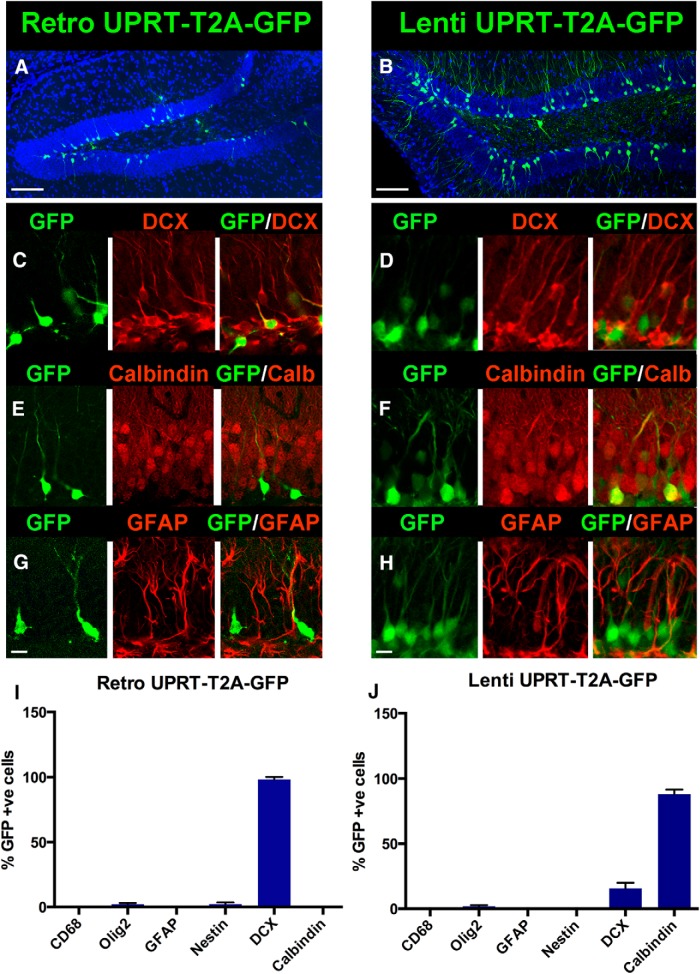
Validation of cell specificity of UPRT-expressing retroviral and lentiviral constructs by immunocytochemistry at 14 DPI. ***A***, ***B***, GFP staining in hippocampal sections from mice injected with UPRT-T2A-GFP retrovirus (***A***) and lentivirus (***B***) 14 DPI. Scale bar, 50 μm. ***C–H***, Immunostaining for retrovirally and lentivirally labeled cells with the immature neuron marker DCX (***C***, ***D***), mature neuronal marker calbindin (***E***, ***F***), and the astrocytic marker GFAP (***G***, ***H***). ***I***, ***J***, Retrovirus-expressing neurons were labeled with DCX, but not mature neuron or glial markers at 14 DPI (***I***), whereas lentivirus-expressing neurons were labeled with the mature cell marker calbindin as well as a small population of DCX (***J***). Scale bar, 10 μm. Data are plotted as the mean ± SD.

To confirm the cellular specificity of UPRT-T2A-GFP virus-mediated transfer *in vivo*, we first analyzed the pattern of GFP expression in mice 2 weeks after stereotaxic injection of retroviral and lentiviral vectors into the dentate gyrus of young adult mice. The expected and observed labeling patterns of GFP-labeled neurons with retroviral or lentiviral constructs are shown in Figure 1*D*. Retrovirally infected GFP^+^ cells at 14 d postinjection (DPI) exhibited typical morphology of adult-generated immature newborn granule cells with cell bodies located in the SGZ and short dendrites terminating in the inner molecular layer (Fig. 1*D*, right top). In lentivirus-injected animals at 14 DPI, GFP^+^ cells had mature neuronal morphologies (Fig. 1*D*, right bottom). The cell bodies of lentivirally labeled cells spanned the GCL with highly arborized dendritic processes extending fully across the molecular layer consistent with mature granule cells. We characterized the phenotypes of the two GFP^+^ populations (Fig. 2*A*,*B*) using double immunolabeling with selective markers for progenitors (nestin, GFAP), immature neurons (DCX), mature neurons (Calbindin, NeuN), and glia (GFAP, CD68, Olig2; Fig. 2*C–H*). As summarized in Figure 2, *I* and *J*, the vast majority of the retrovirally labeled cells (95.3 ± 1.9%) were immature neurons (DCX^+^), whereas only a few cells (2.3 ± 1.2%) were neural progenitors (nestin^+^) or oligodendrocytes (2.2 ± 0.8%; Olig2^+^). None of the GFP^+^ cells were astrocytes or microglia, as assessed by immunostaining with GFAP and CD68, respectively. In contrast, lentivirally transduced GFP^+^ cells labeled with the mature neuronal marker Calbindin (85 ± 3.5%), although a small proportion did express DCX (15.6 ± 4.3%). No colocalization was detected with astrocytic (GFAP^+^) or microglia markers (CD68^+^), and only 1.8 ± 0.9% labeled with the oligodendrocyte marker Olig2^+^. Immunostaining with an antibody against Mac-2, a marker of activated microglia, 2 weeks following both retroviral and lentiviral injections revealed weak labeling only along the needle path, suggesting that there was negligible injury/inflammation caused by the viral vectors or the injection (data not shown). These results indicate that retroviral and lentiviral UPRT-T2A-GFP vectors labeled relatively pure populations of DG immature newborn and mature granule cells in the adult brain *in vivo*.

### Generation of RNAseq libraries

To purify thiol-labeled RNA from UPRT-expressing immature and mature neurons, WT mice were injected with either our retroviral or lentiviral vectors 14 d prior to injection of 4-TU (1.25 µl/1 mm) in the dorsal third ventricle. Sixteen hours later, total and TU-tagged RNA were purified from microdissected dentate gyri. Dentate gyri from 4-TU-treated mice that had not been injected with UPRT viral constructs served as a biotinylation control. RNAseq libraries were generated from three hippocampi (each sample included UPRT plus 4-TU, 4-TU alone, and total RNA input) and subsequently were analyzed in biological triplicates (lentiviral dataset 1 and retroviral dataset 2) or quadruplicates (retroviral dataset 1). Libraries were sequenced to a depth ranging from 25 to 39 million reads. After alignment with mouse genome assembly mm10 with RefSeq gene annotation, each sample had a mapping rate of ∼80% to the reference genome and annotation. The uniquely mapped reads were then assigned to the corresponding transcripts.

**Figure 3.**
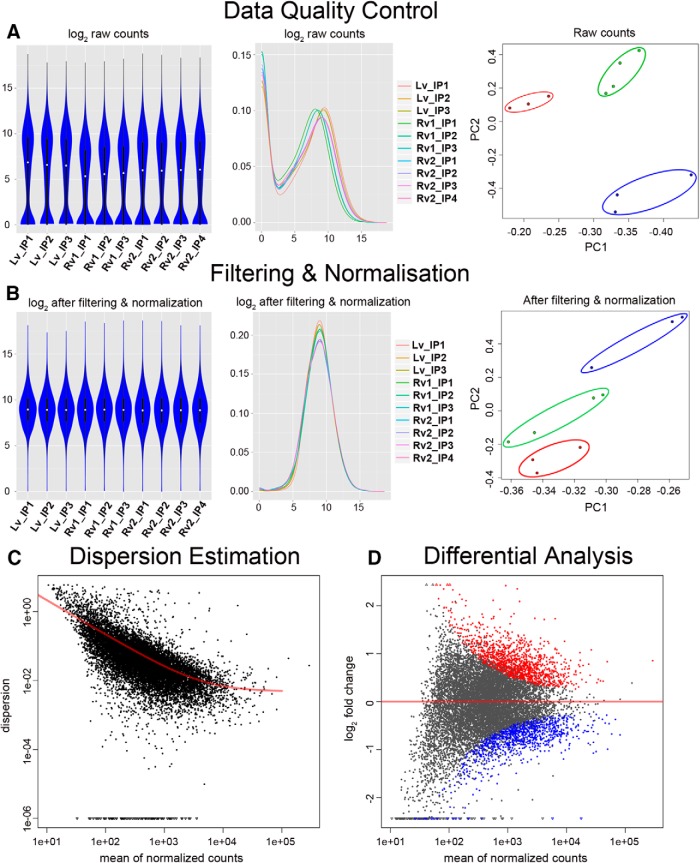
Flowchart for data analysis and quality control of RNAseq. ***A***, The counts assigned to all the genes after mapping were visually checked by violin plot (left; *y*-axis, log reads per gene) and density (middle; *x*-axis, log reads per gene; *y*-axis, number of genes), and by PCA (right). In the PCA plot, the samples clustered into the following three groups: lentiviral dataset is in blue; retroviral dataset 1 is in green; and retroviral dataset 2 is in red. ***B***, The data on counts were subsequently filtered to remove the lowly expressed genes, normalized, and also checked with data shapes and PCA analysis. The color scheme of the three groups in the PCA plot is the same as in ***A***. ***C***, The dispersion of the filtered and normalized data were estimated and compared with the regression line (red). ***D***, The data were tested with a negative binomial model, and ANOVA was used to determine significantly enriched genes and is shown in the M (log ratios) and A (mean average) plot. The red dots depict the significantly enriched genes in the retroviral dataset, and the blue dots depict the depleted genes (i.e., genes enriched in the lentiviral dataset).

The count data examined by violin and density plots showed similar shapes (Fig. 3*A*, left and middle), indicating the consistency of RNAseq libraries across the datasets. Datasets were clustered into three separate groups through PCA (Fig. 3*A*, right). After filtering out very lowly expressed genes and normalization, the data shapes of all datasets were consistent with a Gaussian distribution, whereas the PCA plots retained their corresponding clustering characteristics, indicating that clusters of retrovirus-labeled samples are distinct from lentivirus-labeled samples, suggesting differential expression patterns (Fig. 3*B*). To examine whether there was differential expression of nascent mRNAs between adult born immature neurons 2 weeks postmitosis and mature granule cells, we compared each retroviral dataset to the lentiviral dataset. The dispersion or variance of the count data was estimated and agreed with the regression line, indicating a good fit of our filtered datasets to the binomial statistical model used in DEseq (Fig. 3*C*). The DEseq analysis revealed genes that were significantly enriched in the retroviral (red) and lentiviral (blue) samples. Differential expression was determined using ANOVA for each pairwise comparison, with the cutoff of false discovery rate (FDR) at ≤5% (Fig. 3*D*).

### Differential gene expression analysis in immature and mature granule cells

**Figure 4.**
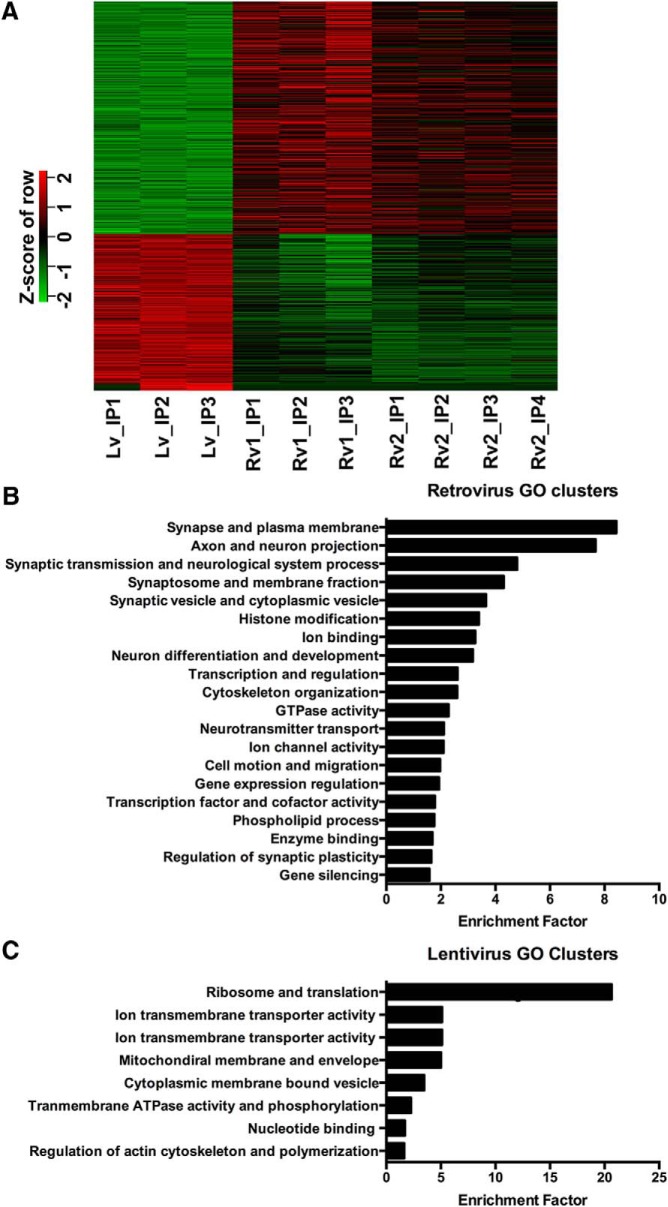
Differential expression of mRNA between immature and mature DG granule cells. ***A***, The retroviral datasets 1 and 2 were separately compared with the lentiviral dataset using a heat map. Each comparison generated a list of significantly changed genes. Both lists were merged into a new single list by requiring that the genes appeared in both lists. The heat map was plotted based on this new list. The 457 genes in the top half of the heat map were specifically enriched in the retroviral datasets. The 305 genes in the bottom half of the heat map were specifically enriched in the lentiviral dataset. ***B***, ***C***, The top enriched ontological clusters of DAVID categories are reported for the retroviral (***B***) and lentiviral (***C***) datasets.

Our experimental strategy using TU tagging allowed us to compare gene expression profiles in immature neurons (∼14 d postmitosis) that have yet to fully elaborate dendrites or receive excitatory synapses with the relatively mature state of neurons at 4 weeks postmitosis. We did not compare the datasets to input (whole dentate RNA) because of the inherent cellular heterogeneity in RNA from the whole dentate. The differential expression of nascent mRNAs was visualized using a heat map (Fig. 4*A*), in which red indicates upregulation and green indicates downregulation. The heat map shows the replicates from the lentiviral dataset with retroviral dataset 1 and dataset 2. There were 457 genes identified as specifically enriched in the retroviral datasets (Fig. 4*A*, top; Table 2, left) and 305 genes specifically enriched in the lentiviral dataset (Fig. 4*A*, bottom; Table 2, right). There was a high degree of consistency between the two retroviral datasets with fold increases (FDR criteria <0.05) that ranged from 1.15 to 7.34. In this comparative analysis, many significantly enriched genes showed only small fold changes (FCs). In the case of the 457 genes in the retroviral dataset, there were 42 in dataset 1 with a fold change of >2, and 106 genes in dataset 2 with a fold change of >2. Because the pulse-labeling strategy with TU-tagging biases our analysis toward nascent RNAs, large fold changes in long-lived RNAs would be masked. This point emphasizes that TU tagging provides different information than transcriptome approaches based on steady-state RNA levels such as qPCR or microarrays.

**Table 2:** Genes specifically enriched in retroviral and lentiviral datasets

457 genes enriched in retroviral datasets							307 genes enriched in lentiviral datasets				
*Gan*	*Psd*	*Phc3*	*Actb*	*Scn3a*	*Rap1gap2*	*Tef*	*Arf1*	*Rps23*	*Csdc2*	*Uqcrc1*	*Gpx1*
*Speer4b*	*Purb*	*C230081A13Rik*	*Swi5*	*Osbpl6*	*Dmxl2*	*Npepps*	*Ube2d3*	*Gdi2*	*2900064A13Rik*	*Tspyl3*	*Fhl2*
*Nova2*	*A930013F10Rik*	*Appbp2*	*Ocrl*	*Efr3b*	*Dlg3*	*Med13*	*Gnb2l1*	*Dars*	*Rps20*	*Aup1*	*Ap2s1*
*Dynll2*	*Tnrc6b+Mir5113*	*Nipbl*	*Nucks1*	*Taok1*	*Ankrd17*	*Pde8b*	*Rpl21*	*Cox4i1*	*Guk1*	*Rps12*	*Ppp5c*
*LOC626693*	*Gm6300*	*Dlg2*	*Epb4.1l1*	*Raph1*	*1810041L15Rik*	*Mtap1b*	*Rpl23*	*Cdk5*	*Hpcal1*	*2410006H16Rik*	*Gpr37l1*
*Lrrc8b*	*Nav2*	*Ash1l*	*6820431F20Rik*	*Jph1*	*Unc80*	*Trim33*	*Atp5a1*	*Yipf5*	*Atp5b*	*Flot1*	*Coro1a*
*Tbrg3*	*Neurl1b*	*Ylpm1*	*Rnf182*	*6430704M03Rik*	*Lrrc4*	*Nsd1*	*Rpl37a*	*Tmem109*	*Rpl18*	*Ifi27l1*	*Ninj1*
*Phxr4*	*Atxn1*	*Pwwp2a*	*Ncam1*	*Wipf3*	*Kif1b*	*Ahcyl2*	*Dbi*	*Prnp*	*Tmem59l*	*Gpc5*	*Gsn*
*Cmah*	*Ncoa6*	*Mll2*	*Arhgef12*	*Slc4a7*	*Dnmt3a*	*Gabrb3*	*Rpl29*	*Tubb3*	*Spag7*	*Dnalc4*	*Elovl7*
*Ppm1l*	*Mbtd1*	*Csmd1*	*Ptpre*	*Camta1*	*Ptp4a2*	*Mgea5*	*Pnkd*	*Sec22b*	*Pdcd6*	*Bex1*	*Sf3b4*
*5031426D15Rik*	*Cdr1*	*Plxna2*	*Ldoc1l*	*Brd3*	*Glg1*	*Atf6*	*Vdac2*	*Psma5*	*Actg1*	*Atp5d*	*Pnpla2*
*Kcnq3*	*C77370*	*Tanc2*	*Itsn1*	*Zdhhc17*	*Gabrb2*	*Sphkap*	*Reep5*	*Gucy1b3*	*Ube2a*	*2610301G19Rik*	*Suclg2*
*Grin2b*	*Olfr613*	*Ranbp10*	*Apc*	*Cnksr2*	*Fyco1*	*Slc1a2*	*Ndufs3*	*Cox8a*	*Tmco1*	*Mrpl49*	*Rbck1*
*1700080N15Rik*	*Ryr2*	*Arhgef9*	*Usp54*	*Chl1*	*Adcyap1r1*	*Rac1*	*Morf4l2*	*Actr1b*	*Tbcb*	*Hk1*	*Spp1*
*Nav3*	*Mll1*	*Ralgapa2*	*Kif21a*	*Ablim1*	*Adcy1*	*Ank2*	*Sparc*	*Tssc1*	*Rps3a*	*Coq6*	*Gadd45g*
*Gatad2b*	*Kbtbd11*	*Larp4b*	*Kif1a*	*Hnrnpd*	*Ip6k1*	*Tnik*	*Hpca*	*Sec11c*	*Ptgds*	*Tmem85*	*Gltscr2*
*Bmpr2*	*Ssbp3*	*Eif2c1*	*Fzd3*	*Hivep2*	*Golgb1*	*Ppp1r9a*	*Rps6*	*Papss1*	*Psmb5+Mir686*	*Rplp1*	*Med18*
*Grin2a*	*Fam178a*	*Atm*	*Srgap3*	*Hipk1*	*Ank*	*Dync1h1*	*Rpl13a+Snord33+Mir5121*	*Dstn*	*Rpl7a*	*Mrpl12*	*Rbp1*
*Arhgap32*	*Tet1*	*Dpp8*	*Srek1*	*Herc2*	*Ube3b*	*Ubr5*	*Ndufa10*	*Ywhaq*	*P4htm*	*Mcrs1*	*Rps10*
*4930470H14Rik*	*Ergic1*	*Setx*	*Ncor1*	*Cblb*	*Rab6b*	*Kpnb1*	*Gad1*	*Rpl27*	*Maea*	*1810063B05Rik*	*Dohh*
*Nup50*	*Vps13c*	*Rmnd5a*	*Klf9*	*Ankhd1*	*Pum1*	*Camk2n1*	*Rps24*	*0610007C21Rik*	*Arl1*	*Snrpb*	*Mib2*
*Onecut2*	*Sobp*	*Malat1*	*Huwe1*	*Myo9a*	*Ccdc88a*	*2610507B11Rik*	*Atp6v1g2*	*Uba2*	*Psmd8*	*Gm12657*	*Tuft1*
*Cdc73*	*Adcy9*	*Fam40a*	*Opcml*	*Sbf2*	*Ankrd12*	*Dsp*	*Rps17*	*Eef1a1*	*Psmd6*	*Sec61b*	*Sulf1*
*Hrk*	*Srrm2*	*Atxn2*	*Lyst*	*Mir770+Meg3*	*Tmem132b*	*Phactr1*	*Rplp2*	*Timm23*	*1110002B05Rik+Mir1892*	*Gpx4*	*Enpp2*
*Gm3893*	*A230073K19Rik*	*Plxna4*	*Gnaq*	*Aldh5a1*	*Snx27*	*Ppig*	*Rpl36*	*Rnf7*	*Txnl1*	*Bsg*	*1500015O10Rik*
*Tbl1x*	*Clmn*	*Nfat5*	*2900026A02Rik*	*Syngr1*	*Scaf11*	*Syn2*	*Rpl35a*	*Pttg1ip*	*Slbp*	*Scrn1*	*Kcnj13*
*Pex5l*	*Tjp1*	*Luzp1*	*Gucy1a2*	*Scn2a1*	*Hecw1*	*B4galt6*	*Rpl24*	*Dynll1*	*Rps27a*	*Puf60*	*Cldn1*
*Mib1*	*Ptprd*	*1810026B05Rik+Chd2*	*Rph3a*	*Herc1*	*Gfod1*	*Zfr*	*Rpl14*	*Aarsd1*	*Prelid1*	*Mlc1*	*Col8a1*
*Palm2*	*Atp6v0a4+D630045J12Rik*	*6230409E13Rik*	*Fat3*	*Arhgap20*	*D430041D05Rik*	*Gria1*	*Pgam1*	*Rps11*	*Fabp5*	*Txndc15*	*Slc4a5*
*Kdm2a*	*Mnt*	*Tacc2*	*9930021J03Rik*	*Smad3*	*Sorbs1*	*Kidins220*	*Npm1*	*Manf*	*Gm12191+Rpl30*	*Chchd2*	*Clic6*
*D10Bwg1379e*	*Eif2c3*	*Ncan*	*Msl1*	*Jhdm1d*	*Scn8a*	*Cbx5*	*Gm12070*	*Ccdc142+Mrpl53*	*Cmpk1*	*Ndufb2*	*Wfdc2*
*Prox1*	*Prrc2c*	*Ntrk3*	*Spire1*	*Thsd7a*	*Dnajc7*	*Slc7a14*	*Atp5f1*	*Eif3i*	*Arxes2*	*Dalrd3*	*Ttr*
*Kcnj6*	*Ipw*	*Dusp18*	*Rsf1*	*Atp8a1*	*Vldlr*	*Hnrnpa2b1*	*Rpl10a*	*Akr1a1*	*Pgrmc1*	*B3gnt1*	*Kcne2*
*A130077B15Rik*	*Fryl*	*Ythdf1*	*Rock2*	*Sppl3*	*Galntl1*	*Zmynd11*	*Ngfrap1*	*Rpsa*	*Rpl3*	*Rpl13*	*Calml4*
*Rnf150*	*1700025G04Rik*	*Elavl3*	*Rev3l*	*Gpd2*	*Scn1a*	*Nell2*	*Micu1*	*Psmd5*	*Coq9*	*Fth1*	*Tmem72*
*Setd5*	*Kcnma1*	*Dscam*	*Anp32a*	*Fam160b1*	*Larp1*	*Trim37*	*Atp6v1g1*	*Ndufs5+BC002163_d*	*up1 Arpc5*	*Tsfm*	*Steap1*
*Nhsl2*	*St3gal2*	*Bod1l*	*Fam63b*	*Fam134a*	*Rb1cc1*	*Sirpa*	*Ndufs2*	*Mrpl15*	*Eif3h*	*Gng10*	*Otx2*
*Tmed9*	*Zfp871*	*Dusp11*	*Agap1*	*Gpatch8*	*Mgat3*	*Cdk14*	*Rpl9*	*Mbp*	*BC003965*	*Dnm2*	*Folr1*
*Igsf3*	*Usp34*	*Ap1g1*	*Whsc1l1*	*Sbno1*	*Cep170*	*Dcaf7*	*Psmd7*	*Flcn*	*9530068E07Rik*	*Adrm1*	
*Crebbp*	*Ube3a*	*Rbfox1*	*Slc38a1*	*Robo2*	*Mtap7d2*	*Sf3b1*	*Ppia*	*Degs1*	*Stmn3*	*Muted*	
*Cnot6*	*Syt7*	*Myt1l*	*Pclo*	*Elavl1*	*Aff4*	*Ptpra*	*Gnb5*	*Caly*	*Cd9*	*Lrrc45*	
*Slitrk3*	*Bsn*	*Lrp1b*	*Nfix*	*Celf1*	*Otub1*	*Stxbp1*	*Gcsh*	*Btf3*	*Adprh*	*Por*	
*Tmcc1*	*Cd47*	*Cadm1*	*Kcna1*	*Agtpbp1*	*Hlf*	*Erc2*	*Cox5b*	*2210016L21Rik*	*Tmsb4x*	*P4hb*	
*Ubn2*	*Bcr*	*Ank3*	*Hook3*	*Zfp238*	*Ganab*	*Armcx5+Gprasp1*	*Atxn10*	*Fam131a*	*Eif6*	*Mgp*	
*Fstl4*	*Cacna1e*	*Ubr4*	*Diras2*	*Smg1*	*Ubqln1*	*Ttc3*	*Rps3*	*2400001E08Rik*	*Eif1*	*Zfp30*	
*Tab3*	*Nav1*	*Rasal2*	*Atg2b*	*Sec16a*	*Nbea*	*Spna2*	*Prdx1*	*Psmc6*	*Dmap1*	*Wls*	
*Dusp8*	*Mdga2*	*Pcdh19*	*Slc4a4*	*Adnp*	*Shisa7*	*Synj1*	*Pgm2*	*Ndufb6*	*Cct4*	*Rps4y2*	
*Tbr1*	*Mdn1*	*Nfib*	*Senp2*	*Tmod2*	*Rasgrf2*	*Nrp1+Mir1903*	*Gm1821_dup1*	*Med28*	*Rabac1*	*Ezr*	
*Dgkh*	*Slco3a1*	*Dstyk*	*Irf2bp2*	*Ppfia2*	*Adrbk2*	*Cyfip2*	*A030009H04Rik*	*Cox6a1*	*Ndn*	*Syt5*	
*Cbfa2t3*	*Map3k3*	*Snx30*	*Ythdc1*	*Pbrm1*	*Arf3*	*Hnrnpu*	*Ugp2*	*Adck3*	*Dusp6*	*Ctso*	
*Dcdc2c*	*Tbcel*	*Mllt3*	*Tcerg1*	*Nktr*	*Trim9*	*Dlgap1*	*Atp6v1h*	*Sucla2*	*Bcl2l1*	*Brp44*	
*Psmd11*	*Atxn7l3b*	*Dpysl2*	*Camsap1*	*Iqgap2*	*Syt1*	*Cttnbp2*	*1500011B03Rik*	*Polr1d*	*Aacs*	*Bex4*	
*Gm17821*	*Sorl1*	*Sema5a*	*Bptf*	*Gm15800*	*Spnb2*	*Tcf4*	*Stx1a*	*Sirt2*	*Rab7*	*Twf2*	
*Foxn3*	*Rc3h2*	*Nfia*	*Neto1*	*Fut9*	*Prkca*	*Prkcb*	*Rps9*	*Sesn1*	*H3f3a*	*Vamp3*	
*Eif2c4*	*Cdc42bpa*	*Fry*	*Foxo1*	*Prickle2*	*Kcnh1*	*Ptk2b*	*Rps7*	*Rpl27a*	*Cnp*	*Nudcd2*	
*Pcdh9*	*Rasgef1a*	*Fam171b*	*Rnf10*	*Fam168a*	*Fam117b*		*Rps25*	*Katnb1*	*Wdr18*	*Lgmn*	
*Fam59a*	*Rc3h1*	*Ccdc82*	*Rfx3*	*Ezh1*	*Atrx*		*Ndrg4*	*Ap1s1*	*S100a10*	*Abcb6*	
*E330033B04Rik*	*Pds5a*	*Mga*	*Pip5k1a*	*Whsc1*	*Nmnat2*		*Hsd17b12*	*Tpt1*	*Phospho2*	*Hgs*	
*Itga4*	*C230091D08Rik*	*Gm10845*	*Fam155a*	*Gcap14*	*Slc24a2*		*Hnrnpk*	*Tomm22*	*Osgep*	*Chchd6*	
*Zfp488*	*Pten*	*Eml5*	*Sv2a*	*Xpr1*	*Olfm1*		*Cfl1*	*Rps13*	*Id3*	*Ubc*	
*Rorb*	*Smg7*	*C330006A16Rik*	*Lsamp*	*Med13l*	*Aak1*		*Acsbg1*	*Rpl7*	*Eif3g*	*Matk*	
*Mll3*	*Prkar2a*	*Setd2*	*Atp2b1*	*Lrrfip1*	*Rbm25*		*Hagh*	*Pebp1*	*Cct6a*	*Ccdc107*	
*Wipf2*	*Odz3*	*Gatsl2*	*Arhgap5*	*Brwd1*	*Negr1*		*Eif4a3*	*Dkk3*	*Nap1l5*	*Hist1h2bc*	
*Nr2c2*	*A630089N07Rik*	*Rod1*	*Trp53inp2*	*Atp2b2*	*Sort1*		*Apbb1*	*Commd8*	*Itm2b*	*Hint2*	
*BC005561*	*Mlec*	*Mycbp2*	*Celf2*	*Akap6*	*Sept3*		*Abcb8*	*Plk2*	*Hadh*	*Haghl*	
*Kcnq1ot1*	*Ablim3*	*Ildr2*	*Cacna2d1*	*Trrap*	*Ubr3*		*Tmeff2*	*Pfkl*	*Ckb*	*Rab1b*	
*Rims1*	*Slc27a4*	*Adam22*	*Abl2*	*Tnfrsf21*	*Mll5*		*Wbp2*	*Nefl*	*Atp5g3*	*2410018M08Rik*	

To examine common functional relationships among the top differentially expressed genes, we performed GO analysis across the molecular function, cellular component, and biological process domains. The top GO clusters for genes differentially upregulated in the retroviral datasets and lentiviral dataset are presented in order of DAVID enrichment score (Fig. 4*B*,*C*). The top GO categories in the retroviral datasets (Fig. 4*B*) were linked with synaptic function (e.g., synapse and plasma membrane, synaptic transmission, synaptic vesicles), neuronal morphology (e.g., cell morphogenesis during neuronal differentiation, axon guidance, and cell motion), and transcriptional regulation (e.g., chromatin organization, histone methylation, and DNA modification). These general patterns are consistent with the early phases of dendritic and synaptic development of newborn neurons at 2 weeks postmitosis (see also Miller et al., 2013). Transcription factors were the single largest category with >65 genes among the 457, only some of which have previously been linked to early neuronal development (Table 2).

At the single-gene level, the differential transcription profile of 2-week-old adult born neurons revealed by our TU-tagging analysis showed a high overlap with transcripts previously linked to adult hippocampal neurogenesis and maturation. For example, transcripts for developmentally regulated proteins used as markers for immature granule cells were significantly upregulated in the following retroviral datasets: *Prox1* (FC, 2.17; *q* = 5.9E-12); *Tbr1* (FC, 1.97; *q* = 0.06); and *Ncam* (FC, 1.71; *q* = 2E-20). Several genes associated with the maturation of immature dentate granule cells were also upregulated, including CREB binding protein (CBP; FC, 2.04; *q* = 2.6E-11), which regulates newborn granule cell development and survival as triggered by GABA-mediated depolarization (Nakagawa et al., 2002; Jagasia et al., 2009). Likewise, *Klf9* (FC, 1.59; *q* = 6.4E-8) acts in terminal stages of neuronal differentiation (Scobie et al., 2009), and *Girdin* (FC, 1.23; *q* = 0.02) regulates axonal development and migration of immature dentate granule cells (Enomoto et al., 2009). These results indicated that these tagged nascent mRNAs are dynamically upregulated in immature newborn granule cells.

At the synaptic level, the retroviral datasets had enrichment of the NMDA receptor subunit gene *Grin2b*, which is expected to be expressed at a higher level in immature (FC, 2.31; *q* = 1E-18)], but *Grin2a* (FC, 1.66; *q* = 0.0019), which shows activity-dependent expression, was also enriched, perhaps indicating that the TU-tagging approach captured the increase in nascent transcripts just as synaptogenesis begins. To link the enriched genes to signaling pathways, we analyzed the enriched genes in the retroviral datasets using the KEGG pathways, which revealed an annotation cluster containing the highest enrichment in the following three terms: axon guidance, Wnt signaling pathway, and long-term potentiation (LTP; see Discussion).

Between 2 and 4 weeks postnatally, dentate granule cells show a dramatic expansion of their dendritic arbors and the formation of excitatory synaptic innervation from the perforant path. Thus, we expected to see differential expression of synaptic genes in the lentiviral dataset, representing mature granule cells, compared with the retroviral datasets, representing immature granule cells. This was not the case. In contrast, the lentiviral dataset showed differential expression in genes associated with metabolic regulation (ribosomes, mitochondria, and oxidative phosphorylation), ion transporters necessary to maintain ion gradient action potential and synaptic activity, and actin polymerization/remodeling, a biochemical process associated with stabilization of the dendritic spines and morphological changes at synapses (Fig. 4*C*). It should be emphasized that synaptic genes are of course expressed in mature cells, but our results are comparative in order to reveal differences in patterns between immature and mature granule cells. Thus, with maturation the enriched genes in mature granule cells reflect the high cellular metabolic demand imposed by the expansion of the dendritic membrane, synapse formation, and increased synaptic activity.

### Comparison of nascent transcript levels obtained by TU tagging with steady-state RNA levels

**Figure 5.**
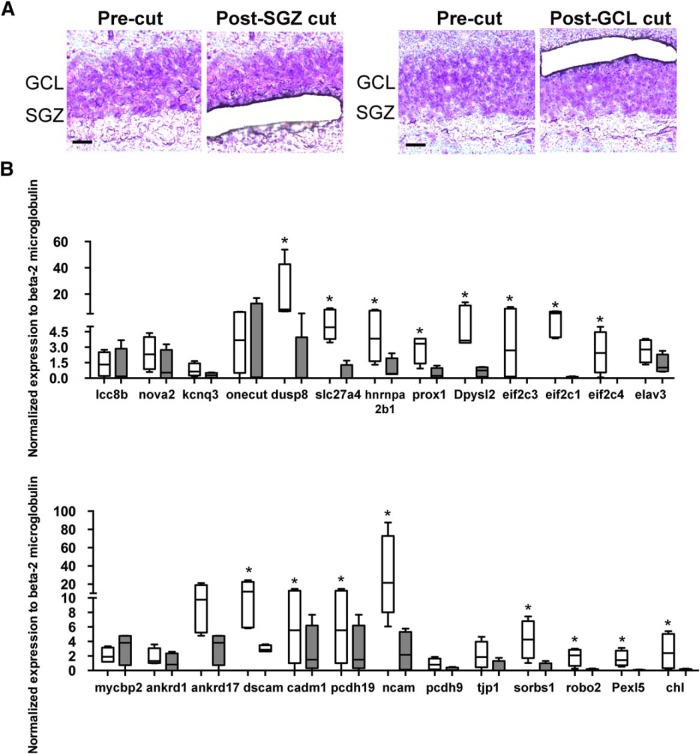
Validation by RT-PCR of TU-tagging target genes in tissue obtained by laser capture microdissection. ***A***, Sequential images of SGZ (two left panels) and GCL (right two panels) show the subregion microdissected by laser capture from cresyl violet-stained dentate gyri. Scale bar, 12 μm. The regions were chosen to show enrichment of immature and mature granule cells, respectively. ***B***, qPCR results from laser-captured tissue showed upregulation in the SGZ (white rectangles) compared with the GCL (gray rectangles) in 62% of the selected genes (16 of 26 genes), mRNA levels were normalized to β_2_-microglobulin. *p < 0.05, *n* = 5).

Two important caveats must be considered in comparing our TU tagging results with traditional transcriptomics. First, TU tagging detects nascent transcripts, which may be distinct from the steady-state mRNA levels obtained by PCR or microarrays. Second, our analysis compared two relatively similar neurons because we were interested in the pattern shifts in gene expression during this period. To compare the RNAseq results with a steady-state RNA method, we combined LCM with qRT-PCR to examine steady-state mRNA levels of selected genes between the subgranular zone, where the majority of the immature newborn cells reside, and the outer margin of the granule cell body layer, which is composed almost entirely of mature granule cells. RNA was isolated from two cell-diameter-wide segments at the inner or outer border of the GCL (representing the SGZ and GCL, respectively; Fig. 5*A*) and amplified for qRT-PCR. mRNA expression levels of 26 genes selected at random from the top GO clusters enriched in the retroviral datasets were analyzed in both SGZ and GCL. Sixteen of the 26 genes (62%) upregulated in the retroviral data were also upregulated (*p* < 0.05) in the SGZ compared with the GCL (Fig. 5*B*). This group included several genes of potential significance for the development of immature neurons, such as transcription factor *Prox1* (*p* = 0.03; Steiner et al., 2008; Lavado et al., 2010; Karalay et al., 2011); cell adhesion molecule Down-Syndrome Cell Adhesion Molecule 1 (*Dscam1*; *p* = 0.03) that has a role in neuronal self-avoidance (Matthews et al., 2007); transcripts for Argonaut proteins (1, 3, and 4; *eif2c1*, *p* = 0.03; *eif2c3*, *p* = 0.03; *eif2c4*, *p* = 0.01) involved in the microRNA-induced silencing complex (Meister, 2013); and roundabout axon guidance receptor homologue 2 (*Robo2*, *p* = 0.03). The detection of differentially expressed genes by both methods implies that these mRNAs show rapid turnover, whereas upregulated genes by PCR, but not TU tagging, are likely long-lived mRNAs.

**Figure 6.**
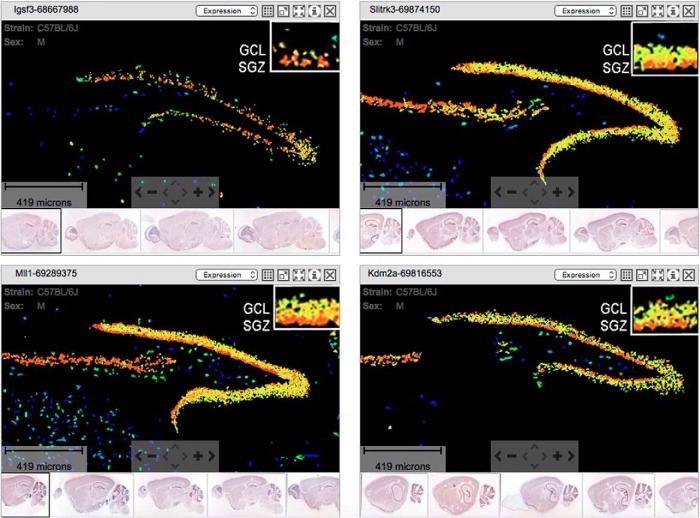
Consistent spatial expression patterns of retroviral enriched genes in mouse SGZ. Representative *in situ* hybridization images from the Allen Brain Atlas for *Igsf3*, *Slitrk3*, *MII1*, and *Kdm2a* show SGZ enrichment, which is consistent with our RNAseq data. The expression energy image display highlights the cells with the highest probability of gene expression using a heat map color scale (from low/blue to high/red).

As a second comparison, we took the top 50 genes from the retroviral dataset and checked the spatial specificity of their expression with *in situ* hybridization results in the Allen Brain Atlas (Fig. 6; images were obtained and modified from http://www.brain-map.org). Of this group, 58% showed the spatial patterns predicted by our RNAseq data (i.e., enhanced expression in the subgranular zone; Fig. 6; a higher expression band occupying the inner border of the GCL), whereas 26% showed expression in both the subgranular zone and the outer margin, and 16% were not detected in the subgranular zone in the atlas. Thus, nascent mRNAs identified in the retroviral dataset showed the proper expression pattern with none expressed in regions with only mature granule cells, confirming the spatial specificity of our analysis.

## Discussion

The idea that changes in gene expression underlie functional alterations in synaptic plasticity, and vice versa, is a central tenet of neuronal development and plasticity. However, the methods used to address these questions with appropriate cell type-specific and high-temporal resolution have been limited until recently. Adult-generated newborn neurons provide a good test of such questions as these cells show definitive stages of morphological and synaptic development over the first weeks postmitosis. We adapted the TU-tagging method to compare profiles of nascent mRNAs in immature and mature dentate granule cells.

### Limitations of technical approach and comparison to prior results

Conventional transcriptome studies (e.g., gene expression microarrays, RNAseq) have been successfully used for gene identification in the regulation of cellular differentiation and other processes. These analyses are based on steady-state mRNA (total mRNA or the mature form of transcripts) by quantifying the abundance of transcripts at a given time, and thus such approaches average across both short- and long-lived transcripts. TU tagging (Cleary et al., 2005, Miller et al., 2009, Gay et al., 2013) can complement these approaches by providing high temporal resolution (16-18 h in our case) as well as cell type-specific resolution. We extended the TU-tagging approach to the intact nervous system to examine nascent mRNA profiles of adult-generated newborn hippocampal granule cells. We used a viral delivery method, which provided superior time resolution (hours rather than days) to transgenic approaches using a floxed UPRT mouse (Gay et al., 2013), and a high degree of certainty that all retrovirus-labeled cells were at the same exact developmental stage. By comparing differential expression between the two viruses, we could also control for any nonspecific changes caused by viral injections. Important to the sensitivity of the TU-tagging approach is the fraction of UPRT^+^ cells compared with the total cells in the tissue sample. Thus, in preparing samples for RNAseq, we microdissected the dentate gyrus so that the URPT^+^ fraction was a few percent, similar to the fractions in prior TU-tagging experiments in endothelial cells (Gay et al., 2013). In the future, the sensitivity of TU tagging may be further improved using methylthiosulfonate-activated biotin for 4SU-RNA isolation to increase efficiency compared with traditional biotin labeling (Duffy et al., 2015).

Other methods have been developed to examine cell type-specific gene profiling (Tallafuss et al., 2014), with each method having specific advantages. For example, translating ribosome affinity purification (TRAP) has the advantage of isolating mRNAs in the process of translation (Sanz et al., 2009). However, TU tagging has potential advantages compared with TRAP because the latter reflects steady-state RNA levels; whereas, TU tagging detects newly synthesized mRNA depending on 4-TU exposure—the shorter the 4-TU treatment, the closer the analysis approximates the transcriptional rate. This feature is important because fold changes in total RNA do not simply reflect alterations in RNA synthesis, but rather depend on differences in transcript turnover. For example, a 20-fold increase in the synthesis of a long-lived transcript (e.g., half-life, 24 h) will cause only a 1.5-fold increase in total RNA after 1 h compared with a >15-fold increase of a short-lived transcript (e.g., half-life, 30 min). Furthermore, TU tagging has the potential to be more sensitive than TRAP because it monitors labeled RNAs directly and also can identify noncoding RNAs.

Cell-specific isolation can also be achieved by mechanical methods such as fluorescence-activated cell sorting (FACS; Wang et al., 2000; Tomomura et al., 2001; Lobo et al., 2006; Cahoy et al., 2008, Bracko et al., 2012) or laser capture (Yao et al., 2005; Rossner et al., 2006; Rohrbeck et al., 2008). For example, laser capture of the subgranular zone in combination with microarrays and *in situ* hybridization revealed genes associated with interneurons, oligodendrocytes, and vascular cells as well as newborn neurons (Miller et al. 2013). The cell damage and time necessary for cell sorting could alter the quality and quantity of RNA, whereas single-cell laser capture can be contaminated by adjacent cells. Methods are evolving for assessing the transcriptomes of single neurons, but gene coverage remains incomplete in most cases (Macaulay and Voet, 2014). By comparison, our retroviral datasets represent an average of 1000–2000 cells.

### Relative gene enrichment in adult-generated immature granule cells compared with mature granule cells

Microarrays have been used extensively to compare differential gene expression in the brain *in vivo* (Lein et al., 2004; Arlotta et al., 2005; Yao et al., 2005; Rossner et al., 2006; Miller et al., 2013; Alldred et al., 2012). Such studies have sometimes revealed genes with large fold increases as a result of a treatment or developmental event (Arlotta et al., 2005; Cahoy et al., 2008; Cho et al., 2013). Thus, we were somewhat surprised that, despite the robust statistical measures of gene enrichment in the retroviral and lentiviral datasets, the fold changes were rather modest. Because we directly compared the retroviral and lentiviral datasets, rather than comparing to a diverse background such as whole dentate RNA, our approach does not provide an absolute measure of RNA transcript levels. This may explain in part why we did not see fold changes of great magnitude. It is also possible that changes in mRNA levels underrepresent changes at the protein level. Unfortunately, there is no direct means to obtain sufficient material of adult-generated immature newborn neurons to directly compare mRNA and protein levels. Furthermore, if the “immature” and “mature” stages overlap, from a biological perspective, then fold changes may be underestimated. Therefore, fold differences may not be the best criteria for evaluating the significance of individual transcripts.

Because TU tagging identifies nascent transcripts rather than steady-state mRNA levels, it is difficult to compare our results directly with prior transcriptome datasets as each provides distinct information. For example, Bracko et al. (2012) used a FACS approach to compare neural stem cells (Sox2^+^) with immature neurons (DCX^+^) with microarray methods. Interestingly, none of their top 10 genes in the DCX^+^ population appears in our top 50 enriched genes in the retroviral dataset of immature neurons. This is not particularly surprising, but it does indicate the complementarity of the two approaches. However, there was some overlap in the GO categories, although it is interesting that more synaptically related categories were detected in our dataset, perhaps indicating that these genes were just beginning to be expressed and thus were detected as nascent transcripts rather than increases in steady-state mRNA levels.

### Patterns of gene expression in newborn neurons

Several interesting patterns emerged from the analysis of the nascent RNAs enriched in the immature neurons, indicating a relative increase in turnover of these transcripts. To assess patterns of known signaling pathways in the retroviral dataset, the following three pathways were most prominent from KEGG analysis: long-term potentiation, Wnt signaling, and axon guidance, with several genes enhanced in newborn neurons in each of these pathways. Nascent RNAs enhanced in the long-term potentiation pathway included *Grin 2a*, *Grin 2B*, *Gria1*, *CBP*, and *PKC* α and β, several of which have known functions in newborn neurons. For example, immature adult-born granule cells express functional extrasynaptic AMPA and NMDA receptors before the onset of excitatory synapses (Tashiro et al., 2006; Tronel et al., 2010; Schmidt-Salzmann et al., 2014). A function for NMDA receptors in adult neurogenesis has been suggested, although their specific role has been debated (Cameron et al., 1995; Nacher and McEwen, 2006). *Grin2a* mutants exhibit deficits in the dendritic morphology of a subpopulaton of dentate granule cells specifically located in the subgranular zone (Kannangara et al., 2014). Similarly, the deletion of *Grin2b* in adult-generated newborn cells did not affect survival, but reduced their dendritic complexity and the pattern separation performance of the transgenic animals (Kheirbek et al., 2012). Enhanced synaptic plasticity of adult-generated granule cells at 1 month postmitosis may also depend on the developmentally regulated expression of *Grin2b* (Ge et al., 2007).

Several members of the canonical (*Frizzled*, *Axam*, *APC*, *Smad3*, *CBP)* and noncanonical (*Prickle*, *Rac*, *ROCK2*, *PKC*, *NFAT*) Wnt signaling pathways were also enriched, which is consistent with their roles in multiple stages of adult hippocampal neurogenesis (Varela-Nallar and Inestrosa, 2013; Wu and Hen, 2013). Blocking Wnt signaling *in vivo* reduced the number of adult-born immature granule cells, and affected hippocampus-dependent spatial and object memory (Lie et al., 2005; Jessberger et al., 2009). Additionally, age-related decline of hippocampal neurogenesis had been associated with dysfunctional Wnt signaling (Miranda et al., 2012). Deletion of *APC* in the adult dentate gyrus caused a reduction of both survival and differentiation of newborn granule cells without affecting their proliferation (Imura et al., 2010). *Smad3* deficiency decreased the survival of newborn DG cells and abolished LTP in the DG, while LTP induction in the CA1 was evoked correctly, indicating a central role for *Smad3* in DG cellular and synaptic plasticity (Tapia-González et al., 2013). In the axon guidance pathway, there were 14 genes that were enhanced in the newborn neurons including semaphorins (*Sema 5A*, *Plexin A*, *NRP1*) and Slit/Robo (*Robo2*) signaling. Although little is known about Robo2 signaling in the dentate gyrus, Semaphorin 3 and 5A have been implicated in granule cell synaptogenesis (Chédotal et al., 1998; Tran et al., 2009; Duan et al., 2014).

In addition to recognizing patterns of expression, gene profiling can provide novel clues to gene function. Among the top 50 enriched genes, as determined by fold increases, only six have known prior functions in newborn neurons, and six others have functions that are likely to be conserved in newborn neurons. Thus, the majority of enriched genes represent targets for further exploration. Overall, the high spatiotemporal resolution of our approach identified an interesting shift in the pattern of gene expression from synaptogenesis and dendritic/axonal development in newborn/immature neurons to an emphasis on the energy demands of synaptic and dendritic maintenance in mature granule cells. In the future, methods to examine turnover of individual transcripts paired with approaches such as TU tagging should provide a more dynamic view of gene regulation and interaction during neuronal differentiation and maturation.
